# 60. Creation and Comparison of a Machine Learning Decision Tree and Traditional Risk Score to Predict Ceftriaxone Resistance in Cancer Patients with *E. coli* Bacteremia

**DOI:** 10.1093/ofid/ofab466.060

**Published:** 2021-12-04

**Authors:** Courtney Moc, William Shropshire, Patrick McDaneld, Samuel A Shelburne, Samuel L Aitken, Samuel L Aitken

**Affiliations:** 1 UT MD Anderson Cancer Center, Houston, Texas; 2 University of Texas MD Anderson Cancer Center, Houston, TX; 3 The University of Texas MD Anderson Cancer Center, Houston, TX; 4 Michigan Medicine, Ann Arbor, TX

## Abstract

**Background:**

There are several clinical tools that have been developed to predict the likelihood of extended-spectrum β-lactamase producing *Enterobacterales*; however, the creation of these tools included few patients with cancer or otherwise immunosuppressed. The objectives of this retrospective cohort study were to develop a decision tree and traditional risk score to predict ceftriaxone resistance in cancer patients with *Escherichia coli* (*E. coli*) bacteremia as well as to compare the predictive accuracy between the tools.

**Methods:**

Adults age ≥ 18 years old with *E. coli* bacteremia at The University of Texas MD Anderson Cancer Center from 1/2018 to 12/2019 were included. Isolates recovered within 1 week from the same patient were excluded. The decision tree was constructed using classification and regression tree analysis, with a minimum node size of 10. The risk score was created using a multivariable logistic regression model derived by using stepwise variable selection with backward elimination at level 0.2. The decision tree and risk score statistical metrics were compared.

**Results:**

A total of 629 *E. coli* isolates were screened, of which 580 isolates met criteria. Ceftriaxone-resistant (CRO-R) *E. coli* accounted for 36% of isolates. The machine learning-derived decision tree included 5 predictors whereas the logistic regression-derived risk score included 7 predictors. The risk score cutoff point of ≥ 5 points demonstrated the most optimized overall classification accuracy. The positive predictive value of the decision tree was higher than that of the risk score (88% vs 74%, respectively), but the area under the receiver operating characteristic curve and model accuracy of the risk score was higher than that of the decision tree (0.85 vs 0.73 and 82% vs 74%, respectively).

Figure 1. Clinical Decision Tree

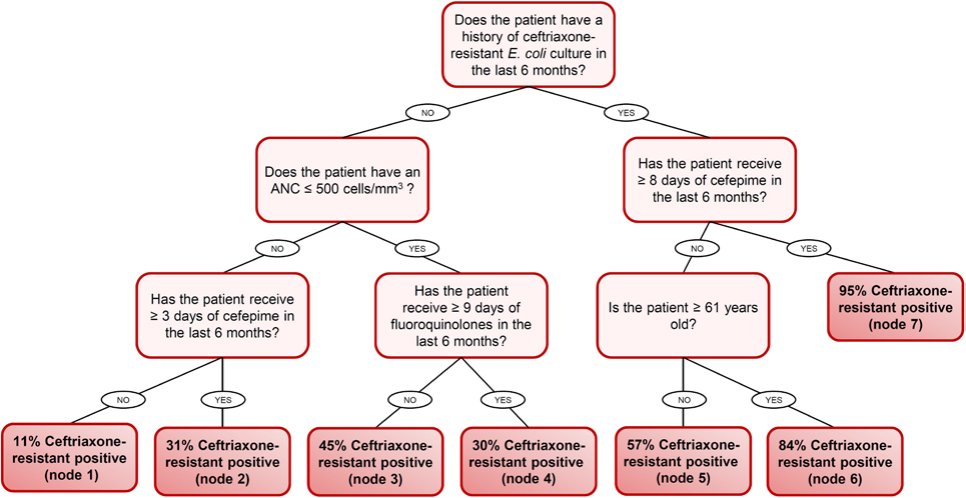

Table 1. Regression Model and Assigned Points for Clinical Risk Score

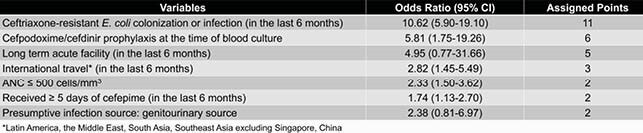

Table 2. Statistical Metrics of Clinical Decision Tree and Clinical Risk Score

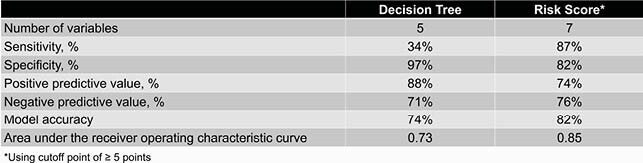

**Conclusion:**

The decision tree and risk score can be used to determine the likelihood of whether a cancer patient with *E. coli* bacteremia has a CRO-R infection. In both clinical tools, the strongest predictor was a history of CRO-R *E. coli* colonization or infection in the last 6 months. The decision tree was more user-friendly, has fewer variables, and has a better positive predictive value in comparison to the risk score. However, the risk score has a significantly better discrimination and model accuracy than that of the decision tree.

**Disclosures:**

**Samuel L. Aitken, PharmD, MPH, BCIDP**, Melinta Therapeutoics (Individual(s) Involved: Self): Consultant, Grant/Research Support

